# The frequent variant A57F in the *GNE* gene in patients from Russia has Finno-Ugric Mari origin

**DOI:** 10.3389/fgene.2024.1511304

**Published:** 2024-12-11

**Authors:** Dmitrii Subbotin, Sofya Ionova, Andrey Marakhonov, Elena Saifullina, Artem Borovikov, Leila Akhmadeeva, Polina Chausova, Oksana Ryzhkova, Rena Zinchenko, Sergey Kutsev, Aysylu Murtazina

**Affiliations:** ^1^ Research Centre for Medical Genetics, Moscow, Russia; ^2^ Department of Neurology, Bashkir State Medical University, Ufa, Russia

**Keywords:** GNE, Nonaka myopathy, distal myopathy, Mari, Bashkir, frequent variant, founder effect, Finno-Ugric people

## Abstract

**Introduction:**

GNE-myopathy is a distal myopathy with adult-onset and initial involvement of anterior leg compartment. A founder effect has been demonstrated for some patients from several large cohorts in different countries.

**Methods:**

In this study, we investigated the allele frequency of the c.169_170delinsTT (p.(Ala57Phe)) variant in the GNE gene (NM_001128227.3) among different ethnic populations (Mari, Tatar, and Bashkir) and estimated the age of the mutation’s spread event.

**Results:**

The c.169_170delinsTT variant in the GNE gene was detected in the Mari population with an allele frequency of 0.003788 but was not found in the Tatar or Bashkir populations. The disease incidence is estimated to be 1.43 (95% CI: 0.00092–43.78) per 100,000 in the Mari population. According to our study, the estimated age of the mutation’s spread is 160.46 years (95% CI: 45.55–244.14).

**Discussion:**

By comparing the information gathered with historical data on migration patterns in the Middle Volga region and estimating the age of the variant’s dissemination, we propose hypotheses regarding its origin and the pathways through which it spread. In the current context of increased rate of interethnic marriages, investigating the spread of common pathogenic variants from historically isolated populations is important for molecular genetic diagnosis. This approach aids in optimizing diagnostic processes and reducing the diagnostic odyssey for patients.

## 1 Introduction

GNE-myopathy (GNEM) is a rare autosomal recessive muscle disease that belongs to the group of distal myopathies with adult-onset initial involvement of anterior leg compartment and caused by biallelic variants in the *GNE* gene (Udd, 2009). GNEM was initially reported by Nonaka et al., in 1981, in 3 patients from 2 Japanese families, as a myopathy with a predominant involvement of the distal limb muscles, and with rimmed vacuoles in muscle biopsies (Nonaka et al., 1981). Later, Argov and Yarom reported it in 9 cases from 4 different Iranian-Jewish families in 1984, as rimmed vacuole myopathy sparing the quadriceps (Argov and Yarom, 1984).

In 2001 and 2002, it was reported that GNEM (Nonaka myopathy, distal myopathy with rimmed vacuoles) is associated with the *GNE* gene, which encoding the bifunctional enzyme, UDP-N-acetylglucosamine 2-epimerase/N-acetylmannosamine kinase (GNE) (Eisenberg et al., 2001; Nishino et al., 2002). This enzyme is the key of the sialic acid metabolism pathway, which limits the rate of biosynthesis of sialic acid and regulates cell surface sialylation (Hinderlich et al., 1997; 2013; Keppler et al., 1999).

Several large cohorts of patients with GNEM have been previously described, and for some patients, a founder effect has been demonstrated (Argov et al., 2003; Nishino et al., 2005; Lu et al., 2011; Chaouch et al., 2014; Mori-Yoshimura et al., 2014; Chamova et al., 2015; Bhattacharya et al., 2018; Chen et al., 2019; Murtazina et al., 2022). The most frequent variants were identified in Middle Eastern Jewish and Bulgarian Roma populations, with the estimated spread age of the mutations reaching up to 1,500 years (Eisenberg et al., 2001; Argov et al., 2003; Chamova et al., 2015).

Recently, a cohort of Russian patients was published uncovering the genetic and clinical spectrum of GNEM in Russia (Murtazina et al., 2022). The study indicated a novel frequent variant c.169_170delinsTT, p.(Ala57Phe), which is clustered among the neighboring Russian regions of Bashkortostan Republic, Mari El Republic and Tatarstan Republic. This variant accounted for approximately 20% of alleles observed in a cohort consisting of 31 patients from 27 unrelated families. This is especially intriguing, given the different historical origins of ethnic groups from these republics. Tatars and Bashkirs are members of Turkic ethnic group indigenous to Russia, while the Mari people are a part of the Finno-Ugric group (Galieva, 2020).

Here, we present a population-based study of the c.169_170delinsTT variant in the *GNE* gene among Russian patients with GNEM from the Mari, Tatar and Bashkir populations.

## 2 Subjects and methods

### 2.1 Patient cohort

**FIGURE 1:**
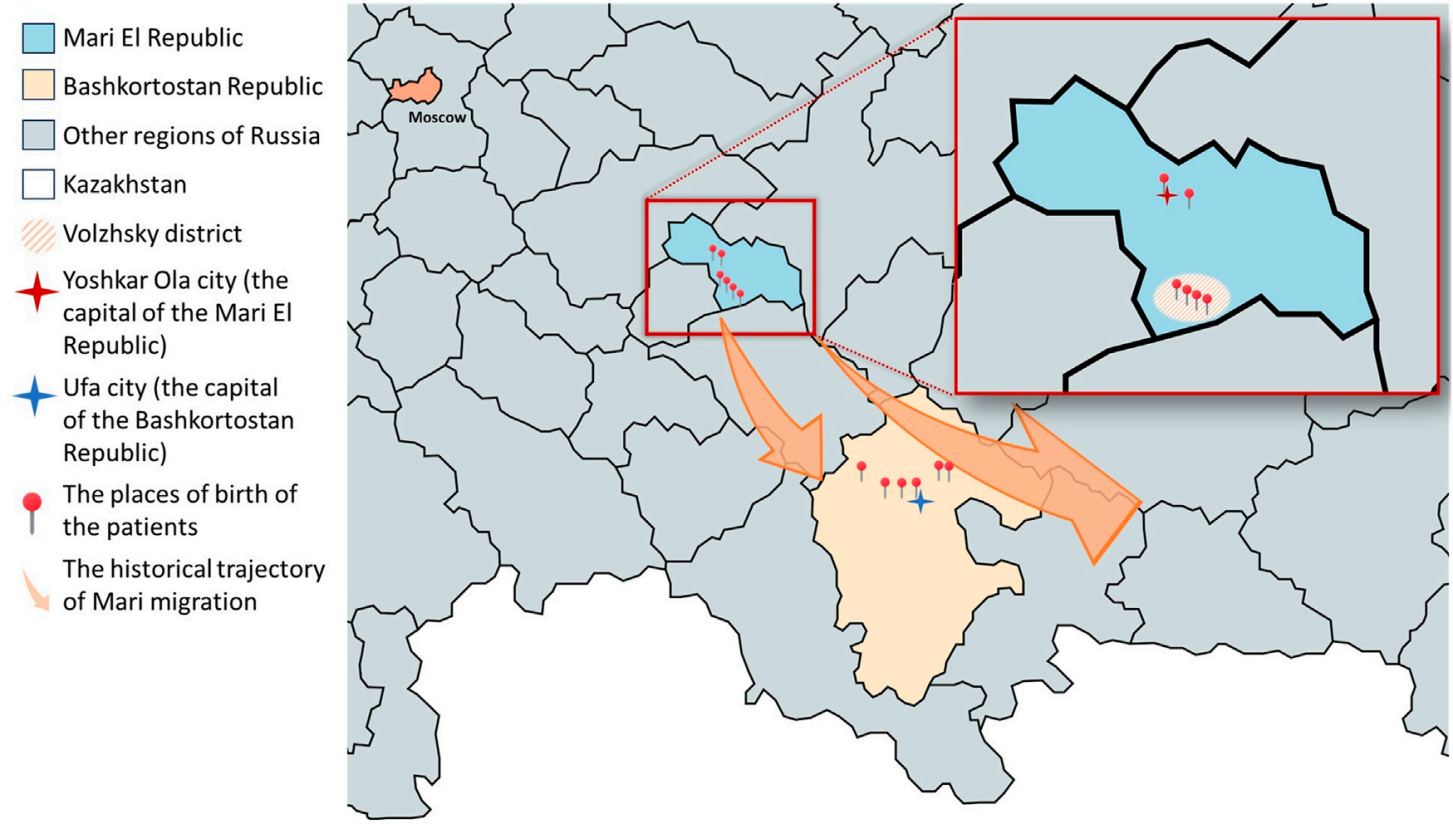
Places of birth of patients with GNEM caused by the c.169_170delinsTT variant in a homozygous state or compound heterozygous state with other variants on the map. The enlarged area shown in the map is the Mari El Republic. The Volzhsky district is highlighted as the place of origin for patients 1.1, 1.2, 1.3, and 6.1. Additionally, the ancestors of patient 4.1, who now resides in Yoshkar-Ola, also originated from the Volzhsky district. Colored arrows indicate the historical trajectory of Mari migration in 16–19 centuries. Created with mapchart.net.

This study included 12 patients from 9 unrelated families, comprising 9 females and 3 males, all of whom had the c.169_170delinsTT variant in the *GNE* gene, either in a homozygous state or as compound heterozygotes with other variants. All patients were born in two regions: the Mari El Republic and the Bashkortostan Republic (Figure 1). Among these patients, three were not included in the previous cohorts reported by Murtazina et al. (2022). The age of these patients at the time of examination ranged from 20 to 44 years, with the disease duration ranging from 2 to 24 years. Eleven of twelve patients were assessed using the GNE Myopathy Functional Activity Scale (GNEM-FAS) (Argov et al., 2017).

We studied historical literature on migration processes in the Middle Volga region and conducted in-depth interviews with probands and their parents to gather detailed family histories. We collected information of at least three generations of studied families.

Patients and their relatives provided written informed consent for the publication of the data.

#### 2.1.1 DNA analysis

For molecular genetic studies, blood samples were collected from the probands, affected and healthy family members. Genomic DNA was extracted using standard methods. For one new patient whole genome sequencing (WGS) was performed, while for two other new patients diagnosis was confirmed by Sanger sequencing.

For WES target enrichment was performed using Illumina TruSeq^®^ ExomeKit (Illumina, San Diego, CA, United States), and custom oligonucleotides (IDT xGen Exome Research Panel v1.0) included coding regions of over 20,000 protein-coding genes. Sequencing was performed using paired-end sequencing (2 × 75 bp) on an IlluminaNextSeq 500 sequencer. The sequencing data were processed using Illumina’s Basespace software (Enrichment 3.1.0). WGS was performed using a DNBSEQ-T7 instrument in a pair-ended mode (2 × 150 b.p.) with an average on-target coverage of 30× with MGIEasy FS PCR-Free DNA Library Prep Set (MGI, Beijing, China) for library preparation (Biotech Сampus Ltd., Moscow, Russia). Sequencing data was processed using the online service for automated bioinformatics analysis of high-throughput sequencing data “NGSData.” The genomic assembly of hg38 was used in the analysis of the detected variants (Borovikov et al., 2024).

Variant annotation was done using Annovar tool (v.2018Apr16). CNV and SV analysis was performed using the Manta tool (v. 1.6.0). Further filtering was performed by functional consequences and population frequencies according to the ACMG recommendations as well as clinical relevance determined by Human Phenotype Ontology database (Köhler et al., 2021).

**FIGURE 2:**
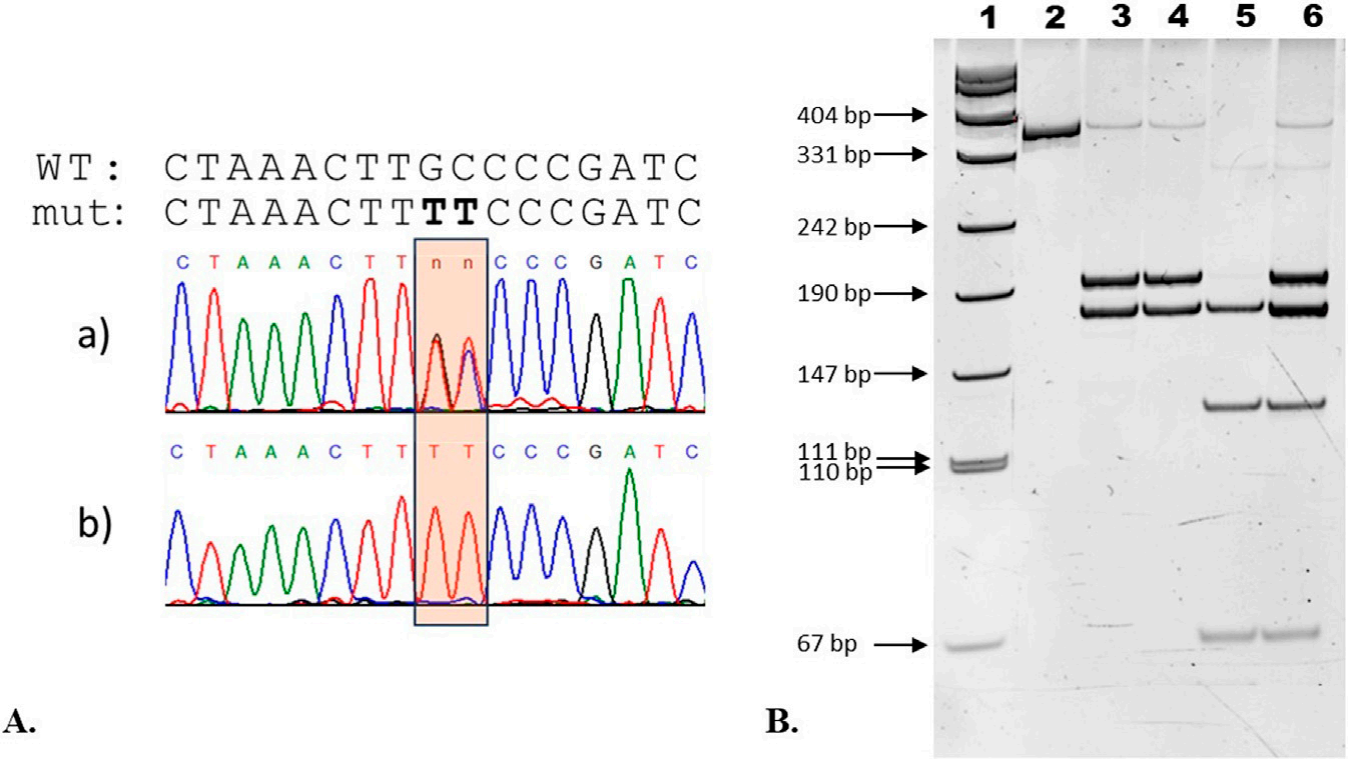
Lanes: 1—pUC19 DNA/Mspl (HpaII) Marker; 2—unrestricted PCR-fragment (357 bp); 3, 4—two products after restriction indicating WT/WT (189 bp, 168 bp); 5—three products after restriction indicating mut/mut (168 bp, 122 bp, 67 bp); 6—four products after restriction indicating WT/mut (189 bp, 168 bp, 122 bp, 67 bp).

The variant c.169_170delinsTT in the *GNE* gene (NM_001128227.3) was directly found in one patient of Bashkir ancestry and was confirmed in all other patients by Sanger sequencing with using specific primers (5′–3′ F:GTGAGAGCCAAGTACCACAACA; R:GGAGAGTCACACATAAGTGGAGGT) (Figure 2). Sanger sequencing was carried out using ABIPrism 3100xl Genetic Analyzer (Applied Biosystems, Foster City, CA, United States) according to the manufacturer’s protocol.

All biological samples have been deposited in the Moscow Branch of the Biobank “All-Russian Collection of Biological Samples of Hereditary Diseases.”

#### 2.1.2 Population study

The allele frequency of nucleotide variant c.169_170delinsTT in the *GNE* gene was assessed in Bashkir population (n = 284) and geographically nearest populations, Mari (n = 132) and Tatar (n = 87), by restriction fragment length polymorphism (RFLP) analysis. RFLP analysis was performed by using restriction enzyme Hpy188III with further analysis of the resulting fragments in 30% polyacrylamide gel (Figure 2). The frequency was calculated and assumed prevalence of the disease was estimated using WINPEPI software (Abramson, 2011). The 95% confidence interval (95%CI) for allele frequency and estimated prevalence was calculated using Klopper-Pearson method.

#### 2.1.3 Autozygosity mapping

Regions of autozygosity on WES and WGS data were analyzed using AutoMap v1.2 tool with the subsequent visual inspection of the regions’ borders (Quinodoz et al., 2021). The age of the mutation event was estimated using the method described in detail elsewhere (Budde et al., 2008; Makretskaya et al., 2023). For the interpolation of hg19 physical positions into the sex-averaged map positions in Kosambi cM, Rutgers Maps v.3 were used (Matise et al., 2007).

## 3 Results

### 3.1 Patient cohort

We report on a patient cohort with GNEM from Russia who had the c.169_170delinsTT variant in the *GNE* gene. Three new patients were added to previously reported patients comprising totally 12 patients from 9 unrelated families with this frequent c.169_170delinsTT pathogenic variant, either in a homozygous state or as compound heterozygotes with other variants (Table 1) (Murtazina et al., 2022). This variant was identified once in a heterozygous state in the control sample of gnomAD version 4.1.0, in a non-Finnish European male, with an allele frequency of 0.0000006196. It is not present in the RUSeq database, a project that consolidates genetic data from clinical laboratories and genomic centers across Russia, based on 6,096 exome samples (Barbitoff et al., 2024).

**TABLE 1:** Genotypes, geographic distribution and general data of the patients from the current study.

Patient No	Gender	Genotype (NM_001128227.3)		Ethnic group	Region of Russia (residence)	Region of Russia (place of birth)	Pedigree peculiarities	Age of examination	Age of onset	Disease duration	GNEM-FAS
		variant 1	variant 2								
1.1	f	c.169_170delinsTT p.(Ala57Phe)	c.169_170delinsTT p.(Ala57Phe)	Mari	Tatarstan Republic	Volzhsky district Mari El Republic	Both parents’ families have resided in this area for the past four generations	30	21	9	73
1.2	m	c.169_170delinsTT p.(Ala57Phe)	c.169_170delinsTT p.(Ala57Phe)	Mari	Tatarstan Republic	Volzhsky district Mari El Republic	No peculiarities	41	26	15	48
1.3	m	c.169_170delinsTT p.(Ala57Phe)	c.169_170delinsTT p.(Ala57Phe)	Mari	Tatarstan Republic	Volzhsky district Mari El Republic	No peculiarities	38	23	15	17
2.1	f	c.169_170delinsTT p.(Ala57Phe)	c.169_170delinsTT p.(Ala57Phe)	Mari	Bashkortostan Republic	Sharan district Bashkortostan Republic	The great-grandfather was of Mari origin	41	20	21	2
3.1	f	c.169_170delinsTT p.(Ala57Phe)	c.169_170delinsTT p.(Ala57Phe)	Mari	Mari El Republic	Medvedevsky district Mari El Republic	No peculiarities	24	17	7	n/d
4.1	f	c.169_170delinsTT p.(Ala57Phe)	c.1853T>C p.(Ile618Thr)	Mari	Mari El Republic	Yoshkar Ola city Mari El Republic	The maternal grandmother was born in the Volzhsky district, Mari El Republic	35	27	8	86
5.1	f	c.169_170delinsTT p.(Ala57Phe)	c.169_170delinsTT p.(Ala57Phe)	Bashkir	Bashkortostan Republic	Blagovarsky district Bashkortostan Republic	No peculiarities	22	18	4	98
6.1	f	c.169_170delinsTT p.(Ala57Phe)	c.169_170delinsTT p.(Ala57Phe)	Mari	Mari El Republic	Volzhsky district Mari El Republic	Both parents’ families have resided in this area for the past three-four generations	20	18	2	78
7.1	f	c.169_170delinsTT p.(Ala57Phe)	c.1652G>A p.(Cys551Tyr)	Tatar	Bashkortostan Republic	Ufa city Bashkortostan Republic	No peculiarities	32	16	16	1
8.1	f	c.169_170delinsTT p.(Ala57Phe)	c.1853T>C p.(Ile618Thr)	Tatar	Bashkortostan Republic	Ufa district Bashkortostan Republic	No peculiarities	39	25	14	48
9.1	f	c.169_170delinsTT p.(Ala57Phe)	c.169_170delinsTT p.(Ala57Phe)	Tatar	Bashkortostan Republic	Karaidelsky district, Bashkortostan Republic	The maternal great-grandmother was Mari origin	42	29	13	46
9.2	f	c.169_170delinsTT p.(Ala57Phe)	c.169_170delinsTT p.(Ala57Phe)	Tatar	Bashkortostan Republic	Karaidelsky district, Bashkortostan Republic	The maternal great-grandmother was Mari origin	44	20	24	19

The frequent variant c.169_170delinsTT p.(Ala57Phe) is highlighted. GNEM-FAS, GNE Myopathy Functional Activity Scale; f, female; m, male, n/d, no data.

Patients with the c.169_170delinsTT variant were examined at the age of 20–44 years and all of them demonstrated typical clinical features, mainly represented by predominantly distal lower leg muscle weakness and atrophy. The duration of the disease in patients ranged from 2 to 24 years. The age of onset was from 17 to 29 years and the first symptoms in most patients were foot drop and/or gait disturbances. As more rare initial symptoms, patient 2.1 exhibited foot drop along with hand weakness, while patient’s 1.1 only symptom was the inability to stand on their toes. Eleven patients were evaluated using the GNEM-FAS, which ranges from 1 to 98 points, where 100 indicates normal functional activity (Argov et al., 2017). Notably, two patients (patients 1.3 and 2.1) with lower GNEM-FAS scores lost ambulatory status 10 years after disease onset, at the age 33 and 30, respectively. Neck muscle weakness was present in 4 out of 9 patients (patients 1.2, 1.3, 2.1 and 5.1). Peculiar findings included facial muscle weakness in patient 5.1 and dysphagia in patient 2.1, who had the most severe clinical status.

In our cohort, 7 patients identified themselves as Mari, 4 as Tatar, and 1 as Bashkir. Among them 6 patients were born in Mari El Republic and 6 were born in Bashkortostan Republic (Figure 1). Most patients from the Mari El Republic were from the Volzhsky district. Three patients from the same family, who identify as Mari, also were born in the Volzhsky district, but currently reside in the Tatarstan Republic (patients 1.1, 1.2, and 1.3). Another patient was from the neighboring Medvedevsky district (patient 3.1), and one from Yoshkar-Ola, the capital of the Mari El Republic (patient 4.1). Interestingly, the grandmother of the patient from Yoshkar-Ola also was born in the Volzhsky district. Among the patients born in the Bashkortostan Republic, one family reported that their great-grandfather was of Mari origin (patients 9.1 and 9.2).

#### 3.1.1 Population study

We analyzed the carrier status of variant c.169_170delinsTT in the *GNE* gene among 503 unrelated healthy inhabitants from three geographically proximate populations, Bashkirs (n = 284), Tatars (n = 87) and Maris (n = 132). Our expectation was to detect carriers of c.169_170delinsTT variant in the *GNE* gene in the Bashkir population. However, c.169_170delinsTT variant was not detected in either Bashkir or Tatar populations. Interestingly, it was identified in one individual in the Mari population (1/132), suggesting a potential high frequency of the families from this population. Allele frequency of variant c.169_170delinsTT in Mari population is 0.003788 (95%CI: 0.000096–0.020923). Based on this data, a disease incidence is estimated to be 1.43480 (95%CI: 0.00092–43.77669) per 100,000 of Mari population.

**FIGURE 3:**
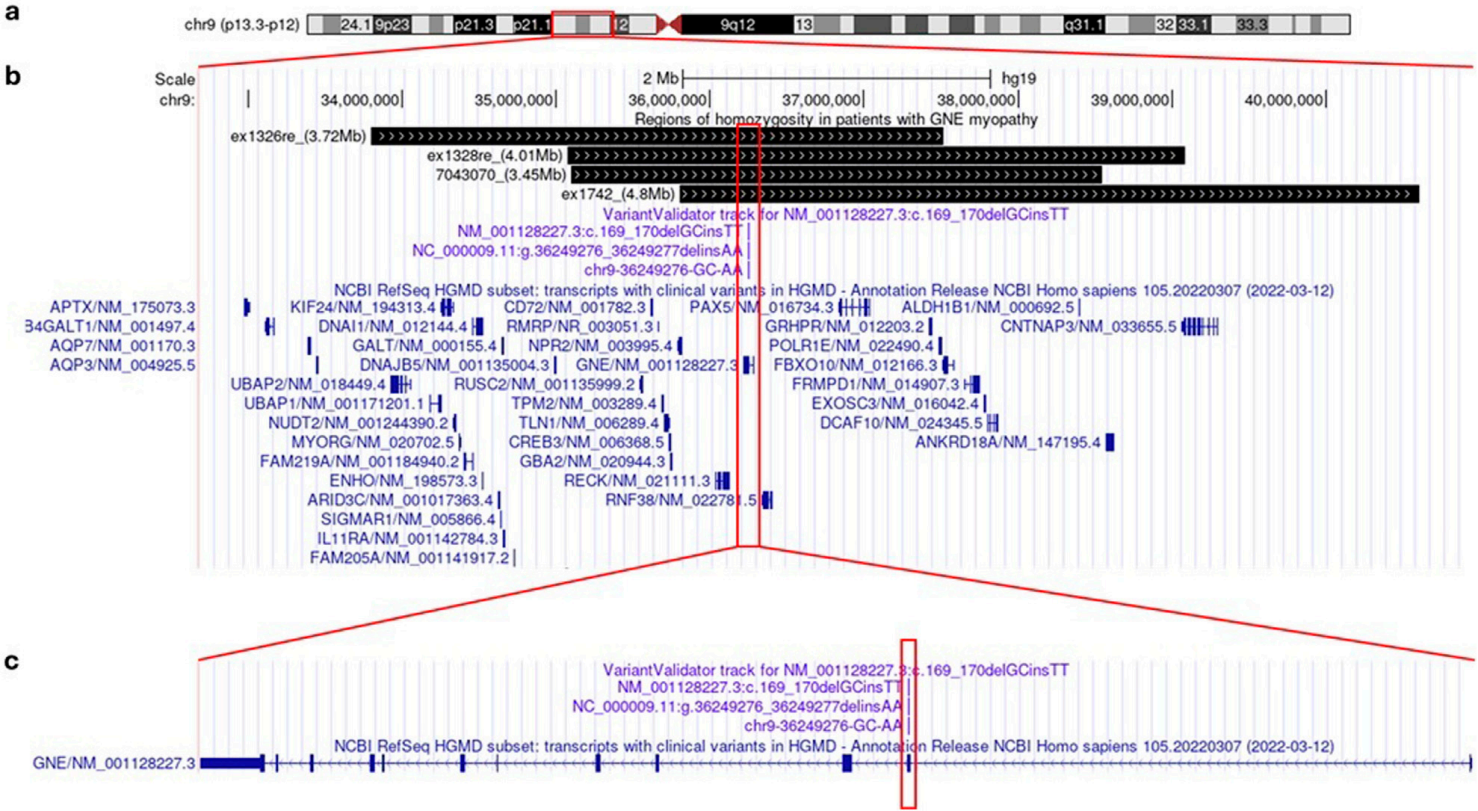
Genetic analysis of GNEM in Mari population in UCSC Genome Browser: ideogram of human chromosome 9 (a); detailed view of regions of homozygosity in patients (depicted in black bars) and NCBI RefSeq HGMD subset of human genes (blue) (b); detailed view of *GNE* gene structure (in dark blue) with NM_001128227.3: c.169_170delinsTT variant location (magenta) (c).

We performed a haplotype study using WES (for 3 cases) and WGS (for 1 patient) analysis in patients with homozygous c.169_170delinsTT variant. As a result, we detected region of homozygosity ranging from chr9:35,096 634–38 543,655 (3.5 Mb) to chr9:35,805 740–40 610,122 (4.8 Mb) encompassing the *GNE* gene (Figure 3).

Using Rutgers Map v.2 (Matise et al., 2007) the physical boundaries of autozygosity regions determined by the WES and WGS data were converted into 2.56–13.72 cM (Kosambi) sex-averaged genetic distances, which corresponds to a common ancestor of 3.90 generations ago (95%CI: 2.56–13.72). Based on the average length of generations in humans of 25 years (Budde et al., 2008), these estimates suggest the age of the mutation spread of 160.46 years (95%CI: 45.55–244.14).

## 4 Discussion

In our study, we conducted a population-based analysis of the frequent variant c.169_170delinsTT in the *GNE* gene among Russian patients with GNEM, which was found in 6 families in homozygous state and in 3 families in compound heterozygous state. Notably, in two unrelated patients, the c.169_170delinsTT variant was found in a compound heterozygous state with the another globally frequent c.1853T>C variant, which had previously been identified in one-third of Russian cases (Murtazina et al., 2022). The c.1853T>C variant has an ancient founder effect in the Bulgarian Roma population, likely originating before the Roma migration from Northwest India around 1,500 years ago (Chamova et al., 2015). This variant accounts for 99% of mutant alleles in that population. Despite the genetic homogeneity of their cohort, the clinical course and severity of the disease vary both inter- and intra-familially. Our two patients with compound heterozygous variants did not exhibit any outstanding features. Additionally, the c.1652G>A variant identified in one of our patients had been previously reported in two unrelated mainland Chinese patients (Zhao et al., 2015).

In the current study, the disease first manifested in the second or third decade of life in all cases. For most patients, the initial symptom was gait disturbance due to predominantly distal muscle weakness and foot drop. In patient 2.1, in addition to gait disturbance, there was also weakness of the distal arms muscles. This patient later developed respiratory issues, dysphagia and loss of ambulation, making it the most severe case in our cohort. It is noteworthy that, despite sharing the same c.169_170delinsTT homozygous variant, patients exhibited varying degrees of disease severity, as reflected in their GNEM-FAS scores. Even within the same family, there was considerable variability: patient 1.3 lost an ambulation at age 33, 10 years after disease onset, while the other two his family members (patients 1.1 and 1.2) showed better outcomes at similar or later stages. It has also been previously reported that the phenotype shows significant intra- and inter-family heterogeneity, even among patients with the same homozygous variant (Chamova et al., 2015; Alrohaif et al., 2018; Mitrani-Rosenbaum et al., 2023). Presumably, the genetic background and environmental factors likely have some effect on the severity and the rate of disease progression.

Previously, it was shown that the frequent variant c.169_170delinsTT in the *GNE* gene is clustered among the neighboring regions of Russia of the Bashkortostan, Mari El, and Tatarstan Republic (Murtazina et al., 2022). In this study, we conducted a more in-depth examination of the history of families living in Tatarstan and discovered that their ancestors actually originate from Mari El. Three new patients with this homozygous variant were also found in the Bashkortostan and Mari El Republics. It is worth noting that, therefore, all patients in our cohort originated from the Republics of Bashkortostan and Mari El (Figure 1).

After analyzing the carrier status of the c.169_170delinsTT variant in the *GNE* gene among unrelated healthy individuals from three different ethnic groups, Bashkirs, Tatars, and Maris, we identified this variant exclusively in the Mari population, with an allele frequency of 0.003788. The estimated incidence rate of the disease in the Mari population is 1.43480 per 100,000. In our cohort, seven patients identified themselves as Mari, which currently reside in Mari El, Bashkortostan and Tatar Republic, four as Tatars, despite the fact that they currently reside in Bashkortostan Republic, and one as Bashkir. Given the predominantly Mari origin of our patients, along with historical data on the migration of the Mari people to Bashkortostan Republic, and absent this variant in the Russian population database RUSeq, we can hypothesize that c.169_170delinsTT variant has a Mari origin (Barbitoff et al., 2024).

Since the proportion of closely related marriages among the Mari population is low (Mamedova et al., 1997), such a high estimated frequency of GNEM in the studied population suggests a founder effect in the distribution of the c.169_170delinsTT variant in the indigenous population. To test this hypothesis, haplotype study in patients with homozygous c.169_170delinsTT variant was performed. We found that the estimated age of the mutation spread was 160.46 years (95%CI: 45.55–244.14). In patients with GNEM, more than 10 different variants exhibiting a founder effect have been identified worldwide, with the estimated spread age of the variant reaching approximately 700 to 1,500 years (Eisenberg et al., 2001; Argov et al., 2003; Chamova et al., 2015; Attri et al., 2021).

In the pedigrees of many of our patients, there is a connection to Mari peoples, including two Tatar patients from Bashkortostan. Additionally, it has been discovered that there are distinct groups of formerly Mari people living in the Republic of Bashkortostan who have been assimilated and now identify themselves as Bashkirs. Based on these data, we hypothesized that a portion of the Mari people have relatively recently migrated from Mari El Republic to neighboring regions and settled there.

It is important to note that while the Republic of Tatarstan borders the Republic of Mari El, Bashkortostan is located farther away (Figure 1). Therefore, the presence of Mari people in Bashkortostan cannot be solely attributed to the geographical proximity of these regions. The Bashkirs are a Turkic people, indigenous to the Southern and partially Middle Urals, Bashkortostan. The Bashkirs became part of the Russian state in the 16th century. Over the next four centuries, the Islamization and adoption of the Tatar language by various peoples facilitated the integration of Tatar and Bashkir cultural elements. This period saw the consolidation of the Bashkirs and Tatars with the Mari, leading to an ethnogenetic unity among these diverse groups (Narody Baškortostana: istoriko-ètnografičeskie očerki, 2002).

According to the 2020 All-Russian Population Census, there are 423,803 Mari people living in Russia. Of these, 246,560 reside in the Mari El Republic, 84,988 in the Republic of Bashkortostan, and only 15,666 in the Republic of Tatarstan. The Mari are a Finno-Ugric ethnic group primarily residing in the Middle Volga region, with the majority located in the Mari El Republic. Compact groups of Mari also inhabit other regions, including the Republics of Bashkortostan and Tatarstan. The Mari began settling these territories in the 16th and 17th centuries. The 16th-century military conflict with the Kazan Khanate accelerated their migration to the Bashkir Urals. The peak of Mari migration to Bashkortostan occurred between the late 17th and early 18th centuries. This migration was driven by several factors: the peasant war of 1,670–1,671, increased taxation, and forced Christianization in the Middle Volga region. Additionally, the agricultural conditions in Bashkortostan resembled those in the Volga region, and the local Muslim population exhibited a more tolerant attitude toward the pagan Mari people. These factors laid the groundwork for significant cultural integration of the Mari people with other ethnic groups in the Republic of Bashkortostan. Over time, the ethnic identity of Finno-Ugric immigrants increasingly shifted toward a Bashkir identity, largely due to intermarriage with the Bashkirs (Rachmatullin, 1988; Akmanov, 1991; Narody Baškortostana: istoriko-ètnografičeskie očerki, 2002; Sadikov, 2016; 2023; Galieva, 2020). We highlight this fact because at least one Bashkir family in our cohort reported having Mari ancestry. Compared to the 18th century, the Mari population has grown sevenfold (Jurkov and Russland, 1998). This significant growth over three centuries, along with limited migration, may have led to the preservation of certain genotypes.

Forced Christianization continued actively in these territories throughout the 18th century (Sadikov, 2016; 2023). Although mass migrations of the Mari people to Bashkortostan were not reported in the 19th century, we assume that migration processes continued on a smaller scale due to ongoing negative political and social events.

We identified a homozygous c.169_170delinsTT variant in two unrelated families from the Volzhsky district, one family from the neighboring Medvedevsky district Mari El Republic, and two Bashkir families from the Sharansky and Blagovarsky districts Bashkortostan Republic. These districts in Bashkortostan Republic are historically inhabited by former Mari people (Narody Baškortostana: istoriko-ètnografičeskie očerki, 2002). These findings, in conjunction with the historical context, suggest a founder effect within the Mari population, likely originating in the Volzhsky district. The c.169_170delinsTT variant may have subsequently spread to nearby territories and, through migration processes, reached the more distant Bashkortostan.

The limitation of our study included the small number of patients with the c.169_170delinsTT homozygous variant who underwent WES or WGS to estimate the predicted age of the mutation. Additionally, we had a limited number of samples from healthy Mari populations, which restricted our ability to precisely estimate the allele frequency of this variant.

## 5 Conclusion

In this study, we build on previous research by investigating the origin and frequency of the c.169_170delinsTT variant in the Mari and Bashkir populations. We describe the phenotypic spectrum for 12 patients from 9 unrelated families who share this common variant. Additionally, our in-depth analysis of population history and the determination size of locus autozygous help us link this variant with the Mari origin and estimate the spread age of the variant reaching. Understanding the accumulation of frequent pathogenic variants in populations is crucial for optimizing molecular genetic testing and providing a more individualized approach to molecular diagnostics.

## Data Availability

The datasets presented in this article are not readily available because of patient privacy. Requests to access the datasets should be directed to the corresponding author.
